# Getting the Right Clones in an Automated Manner: An Alternative to Sophisticated Colony-Picking Robotics

**DOI:** 10.3390/bioengineering11090892

**Published:** 2024-09-03

**Authors:** Lorena Hägele, Brian F. Pfleger, Ralf Takors

**Affiliations:** 1 Institute of Biochemical Engineering, University of Stuttgart, 70569 Stuttgart, Germany; lorena.haegele@ibvt.uni-stuttgart.de; 2 Department of Chemical and Biological Engineering, University of Wisconsin-Madison, Madison, WI 53706, USA; brian.pfleger@wisc.edu

**Keywords:** laboratory automation, strain construction, high-throughput clone selection, colony-picking station, biofoundry

## Abstract

In recent years, the design–build–test–learn (DBTL) cycle has become a key concept in strain engineering. Modern biofoundries enable automated DBTL cycling using robotic devices. However, both highly automated facilities and semi-automated facilities encounter bottlenecks in clone selection and screening. While fully automated biofoundries can take advantage of expensive commercially available colony pickers, semi-automated facilities have to fall back on affordable alternatives. Therefore, our clone selection method is particularly well-suited for academic settings, requiring only the basic infrastructure of a biofoundry. The automated liquid clone selection (ALCS) method represents a straightforward approach for clone selection. Similar to sophisticated colony-picking robots, the ALCS approach aims to achieve high selectivity. Investigating the time analogue of five generations, the model-based set-up reached a selectivity of 98 ± 0.2% for correctly transformed cells. Moreover, the method is robust to variations in cell numbers at the start of ALCS. Beside *Escherichia coli*, promising chassis organisms, such as *Pseudomonas putida* and *Corynebacterium glutamicum*, were successfully applied. In all cases, ALCS enables the immediate use of the selected strains in follow-up applications. In essence, our ALCS approach provides a ‘low-tech’ method to be implemented in biofoundry settings without requiring additional devices.

## 1. Introduction

In recent years, the basic concept of the design–build–test–learn cycle (DBTL cycle) has been continuously upgraded to streamline the process of efficient strain construction and evaluation [1]. Modular design tools, data management systems, and models were integrated in the DBTL cycle to support the first step of design [2,3]. The build part, which predominately comprises DNA assembly, molecular cloning, and host transformation, is steadily optimised by embedding novel genetic-engineering tools which may even be automatable [4,5,6,7]. By analogy, subsequent strain testing, which is conventionally conducted in manual batch cultures using shaking flasks, mL, or µL devices, is often transferred into automated routines. Still, essential readouts for qualifying strain performance, such as growth product formation and strain robustness, are analysed [8,9]. After all strains have been tested, learning is a crucial step of the DBTL cycle. Besides conventional statistical evaluation, model-guided performance checks [10] and machine-learning tools [11] are studied. The DBTL cycle enables the application of high-throughput methodologies [12], thereby screening an abundance of different constructs. The rigorous implementation of fully automated DBTL cycles, the so-called biofoundries [13,14], are gaining momentum as an essential pillar of synthetic biology in academia [15] and in industry [16,17,18].

In the building step, processes such as DNA construction, molecular cloning, and transformation can be effectively executed using liquid-handling techniques [14,19]. Modern techniques such as DNA construction using high-fidelity DNA polymerases and molecular cloning techniques like Golden Gate assembly exhibit a minimum error rate and high robustness [20,21]. For example, when assembling a backbone and a single insert through Golden Gate assembly, a cloning efficiency of 100% is commonly achieved [21]. However, a significant bottleneck arises during clone selection. In traditional laboratory settings, this step involves the application of transformed cells onto solid agar plates, followed by incubation, and subsequent manual selection of individual colonies for further analysis. Colony picking is conventionally performed manually or in a semi-automated manner [22,23,24], which makes it time-consuming and susceptible to errors. Also, biofoundries may make use of automated colony-picking stations [19,25,26,27,28]. Key performance problems remain, such as the potential rejection or failure to pick colonies correctly because of overlapping neighbouring colonies [29], the impact of different agar heights on the robot [30], and the intrinsic quality dependency on the system specification [31]. Some customised solutions, applying a combination of liquid-handling robots and charge-coupled device (CCD) cameras, have been proposed [32,33]. However, the cost and complexity of such colony-picking stations is challenging for academic biofoundries.

To face this issue, various clone selection methods have been implemented in (semi) automated platforms designed for DBTL cycles. They are challenged by the ultimate goal of achieving the highest selectivity with the maximum speed, only requiring minimum complexity. As different biofoundries show, the application of polyclonal screening offers rapid progress but may suffer from reduced selectivity [5,6,34]. Alternately, flow cytometry may be integrated in the cell selection step [35] to support the cell-gating process by additional information about, e.g., cell size distribution and cell-specific labelling. For the latter, the information given by proper biosensors is essential. Recent developments make intensive use of microfluidics for clone selection. The single-cell microliter-droplet screening system, known as MISS Cell, was designed for clone selection and cultivation [36].

From the perspective of a low-budgeted wet lab, almost all approaches possess the intrinsic drawback of incorporating additional devices. The only exception is the polyclonal selection but for the sake of poor selectivity. Consequently, our primary goal was to develop a universally applicable liquid-handling method that may be applied in any biofoundry without further investment. In developing the method, we focused on a selection technique superior to polyclonal screening, capable of generating production strains following DNA assembly. Besides *Escherichia coli* (*E. coli*), the current study focuses on *Pseudomonas putida* (*P. putida*) and *Corynebacterium glutamicum* (*C. glutamicum*) to showcase our automated selection approach. The model-based procedure successfully makes use of the uniform growth behaviour of the correctly transformed cells within the clone selection. The boundaries of experimental applicability are investigated.

## 2. Materials and Methods

### 2.1. Media and Buffer Solutions

A BHI medium was prepared by adding 37 g/L BHI (Becton, Dickinson and Company, Franklin Lakes, NJ, USA), and a 2xTY medium was prepared by adding 10 g/L yeast extract, 16 g/L tryptone, and 5 g/L NaCl dissolved in deionised water. For agar plates, 18 g/L of agar–agar was added prior the autoclavation. The BHIS medium contained 37 g/L of BHI (Becton Dickinson GmbH, Heidelberg, Germany) and 91 g/L of Sorbitol dissolved in deionised water. An SOC medium was prepared by adding 5 g/L yeast extract, 20 g/L tryptone, 10 mM NaCl, 2.5 mM KCl, 10 mM MgCl_2_, and 10 mM MgSO_4_ dissolved in deionised water. After autoclavation, glucose was added to a final concentration of 20 mM. Appropriate antibiotics were added to the liquid medium as well as to the agar plates in the following concentrations: ampicillin 100 µg/mL and kanamycin 50 µg/mL.

Buffers for preparation of electro competent cells were prepared by adding 25 mL glycerol and 20 mL 0.5 mM HEPES buffer (pH 7.2) dissolved in 500 mL of deionised water (EPB 1) and by addling 30 mL of glycerol and 2 mL of a 0.5 mM HEPES buffer (pH 7.2) dissolved in 200 mL of deionised water (EPB 2). The PBS buffer contained 10 mM Na_2_HPO_4_, 137 mM NaCl, 2.7 mL KCl, and 1.8 mM KH_2_PO_4_ dissolved in deionised water and adjusted to pH 6.8. Cryoprotective solution was prepared by adding 15% glycerol to PBS buffer.

### 2.2. Bacterial Strains and Plasmids

*Escherichia coli* BL21 (DE3), *Pseudomonas putida* KT 2440, and *Corynebacterium glutamicum* ATCC 12032 were used for the automated liquid clone selection. The strains *P. putida* and *C. glutamicum* were cultivated at a temperature of 30 °C, while *E. coli* was cultivated at 37 °C. For transformation, different plasmids were used based on the different organisms. The plasmid pJOE4056.2 was transformed into *E. coli*, pJOE7706.1 was transformed into *C. glutamicum*, and pWN210 was transformed into *P. putida*.

### 2.3. Transformation Procedure for E. coli BL21 (DE3), P. putida KT 2440, and C. glutamicum ATCC 12032

To obtain chemically competent cells, *E. coli* BL21 (DE3) and *P. putida* KT 2440 were cultured in 5 mL of the 2xTY medium at 37 °C on a rotary shaker set to 130 rpm for 16 h to 20 h. For main cultures, a 200 mL 2xTY medium in a 1 L baffled-shaking flask was inoculated with 500 µL of the preculture and cultivated at 37 °C on a rotary shaker set to 130 rpm. Upon reaching an OD_600_ of 0.5, the cells were harvested by centrifugation at 5000 rpm for 10 min at 4 °C. The supernatant was discarded, and the cells were resuspended in 15 mL of 0.1 M CaCl_2_. This was followed by an additional centrifugation step, as described previously. The washing process was repeated twice, and finally, the cells were resuspended in 2.5 mL of 0.1 M CaCl_2_ containing 15% glycerol. Aliquots of 200 µL were frozen in liquid nitrogen and stored at −70 °C.

For the generation of electrocompetent cells, *C. glutamicum* ATCC 12032 was cultured in a 5 mL BHIS medium at 30 °C on a rotary shaker set to 130 rpm for 16 h to 20 h. For the main cultures, a 100 mL BHI medium in a 500 mL baffled-shaking flask was inoculated with 1 mL of the preculture and cultivated at 30 °C on a rotary shaker set to 130 rpm. Upon reaching the OD_600_ of 0.6, ampicillin was added to a final concentration of 1.5 µg/mL, and cultivation continued for 1 h. Cells were harvested by centrifugation at 5000 rpm for 7 min at 4 °C. After discarding the supernatant, the cells were resuspended in 30 mL of EPB 1, followed by an additional centrifugation step, as previously described. The washing process was repeated three times, and the cells were finally resuspended in 2 mL of EPB 2. Aliquots of 600 µL were frozen in liquid nitrogen and stored at −70 °C.

Glycerol stocks of the strains containing the desired plasmid were used to inoculate 5 mL of the 2xTY medium. The tube was incubated at 37 °C on a rotary shaker set to 130 rpm for 16 to 20 h. Plasmids were extracted using an E.Z.N.A. Plasmid DNA Mini Kit I (omega BIO-TEK, Norcross, GA, USA) according to the manufacturer’s instructions. Plasmid DNA was normalised to 20 ng/µL.

Heat shock transformation involved mixing 45 µL of chemical competent cells and 5 µL (100 ng) of desired plasmid DNA (Table 1), followed by incubation on ice for 30 min. The transformation approach was then incubated at 42 °C for 60 s. After heat shock, the transformation approaches were again incubated on ice for 2 min. The transformants were recovered in SOC medium for 1 h at 37 °C on an incubation shaker set to 800 rpm. Prior to the development of the ALCS method, this protocol was optimised (see Supporting Information S3).

For transformation using electroporation, 150 µL of electrocompetent cells were mixed with 5 µL (100 ng) of the desired plasmid DNA. The transformation approach was then transferred to an ice-cold electroporation cuvette, and electroporation was carried out with the following settings: 250 µFD, 200 Ω, 25 µF, and 2.5 kV for 4 to 5 ms. The transformants were recovered in a prewarmed BHIS medium for 6 min at 46 °C on an incubation shaker. The transformation approaches were cultivated at 30 °C on an incubation shaker set to 800 rpm for 1.5 h.

An aliquot of 50 µL of the regenerated transformed cells was either plated on agar plates containing the appropriate antibiotics or used for inoculation of the automated clone-selection procedure using the automated microbioreactor device.

### 2.4. Evaluation of the Transformed Cells on Agar Plates

A volume of 50 µL from the transformation approach was inoculated into three wells of a 6-well agar plate containing the appropriate antibiotics. The plates were cultivated at 37 °C for 25 h for *E. coli*, at 30 °C for 69 h for *P. putida*, and at 30 °C for 50.5 h for *C. glutamicum*. Images were captured from the plate subsequently at regular intervals. Those images were analysed using ImageJ [40]. The relative area of the colony-forming units (CFU), in relation to the agar plate itself, was calculated assessing growth at different time points. The estimation of cell number (Equation (1)) was based on the average size of an *E. coli* cell (2.2 × 10^−6^ mm^2^) [41], an *P. putida* cell (2 × 10^−6^ mm^2^) [42], and *C. glutamicum* cell (2 × 10^−6^ mm^2^) [43]. Using these growth parameters, the growth rate (µ, Equation (2)) and generation time (Equation (3)) were calculated:

(1) $$cell\;number\;\left[abs.\right]=\frac{area\;CFU\;\left[{mm}^{2}\right]}{area\;average\;size\;microorganism\;\left[{mm}^{2}\right]}$$

(2) $$µ\;\left[h^{-1}\right]=\frac{\ln\left({cell\;number}_{T1}\right)/\ln\left({cell\;number}_{T2}\right)}{time\;\left[h\right]}$$

(3) $$\mathrm{generation}\;\mathrm{time}\;\left[h\right]=\frac{\ln\left(2\right)}{µ\;\left[h^{-1}\right]}$$

### 2.5. Set-Up of the Automated Liquid Clone Selection (ALCS)

ALCS yields to select a uniform population of correctly transformed clones by harvesting at a distinct growth rate which is characteristic for the modified strain. In essence, growing populations were transferred from one well to the other installing a pre-calculated time of cultivation. The latter was estimated with a growth model (see results), assigning each well a cultivation time equal to the respective generation time of the organism under consideration (see results of transformed cells on agar plates). For clone selection, the disparate growth rates between transformed (indexed ‘1’) and non-transformed cells (indexed ‘2’) were exploited. In essence, missing the antibiotic resistance in non-transformed cells they were anticipated to show a non-survival rate of −0.7 h^−1^ in the antibiotic-containing selection medium. Hence, the ratio of antibiotic-resistant transformants compared to non-transformed cells steadily improved. Although being a preliminary assumption, observations re-assured the initial settings. Off-line flow cytometry was applied to identify the percentage of positively selected clones. The entire selection duration was estimated via the model in Equation (10), considering the upper limit of a proportion of 0.02 non-transformed cells in the last well. The workflow was realised using the automated microbioreactor system, as elaborated upon in the following sections. ALCS is independent of the microbioreactor system used in this study and can be elaborated in any liquid-handling system equipped with a heat-shaker.

Further simplifying procedures were established for the non-*E. coli* strains: Based on the experience with *E. coli*, the growth rates of other transformed hosts (µ_1_) were measured on agar plates. Therefore, the non-survival rate of non-transformed cells was set at −2.8 times µ_1_, in analogy to the *E. coli* case.

### 2.6. Microbioreactor Cultivation for ALCS

ALCS made use of a microbioreactor system (RoboLector L, Beckman Coulter, Brea, CA, USA) equipped with 48-well microplates (FlowerPlate, MTP-48-B, Beckman Coulter, Brea, CA, USA) with a maximum volume of 1 mL of the 2xTY or BHI medium per well. Cultivations were carried out with a humidity of 85%, either at 37 °C or 30 °C, depending to the cultivated microorganism. The shaking frequency was either set to 1100 rpm for *E. coli* and *P. putida* or set to 900 rpm for *C. glutamicum*. To maintain sterile conditions, the plate was sealed with a gas permeable membrane (AeraSeal BS-25, Excel Scientific, Victorville, CA, USA). The backscatter value and the enhanced green fluorescent protein (eGFP) expression were detected via the microbioreactor system.

The current ALCS followed the strategy to install selection steps of one generation time per stage. Only the first selection step comprised the period of two generation times for additionally considering the initial lag phase of the cells. Notably, this setting is somewhat arbitrary and may be adapted for other applications. However, it enabled proper processing, with respect to sampling times, maximum cell densities, and fluid handling. The selection started by inoculating 50 µL of the transformed population into 950 µL of the 2xTY or BHI medium. After two generation times, 500 µL of this first well were transferred by the liquid-handling robot to the next well. Hence, the start concentrations of the subsequent step were half of the final titres in the preceding stage. The procedure continued for four wells, i.e., three transfers. Each well is referred to as one stage of the selection procedure. In case of the Gram-positive *C. glutamicum,* an additional resting time was added to the first stage.

Furthermore, the impact of dilution on the transformation efficiency was investigated. Therefore, a reference curve was generated as follows: First, *E. coli* was transformed as previously reported. After recovery in SOC media, the transformation approach was diluted in SOC media using different dilution steps (1:2 to 1:500). The ALCS was then inoculated with 50 µL of the diluted transformation approach.

For further analysis, the growth rates of the different stages were calculated (see Equation (2)), and single-cell analysis was carried out.

### 2.7. Single-Cell Analysis of the Clone Selection Procedure

Samples from the ALCS were automatically collected after each transfer using the liquid-handling system. A volume of 200 µL of the broth was centrifuged at 13,000 rpm at 4 °C for 5 min and then resuspended in cryoprotecting solution. Samples were stored until analysis at −20 °C. The stored samples were thawed on ice and centrifuged again at 13,000 rpm at 4 °C for 5 min to remove the cryoprotecting solution. Subsequently, the samples were resuspended in 100 µL of PBS. If the samples had an OD_600_ above 0.2, they were diluted to a final OD_600_ of 0.2. Both the wildtype strain *E. coli* BL21 (DE3) and the monoclonal culture of *E. coli* BL21 (DE3) containing the plasmid pJOE4056.2 were used as a negative and positive control, respectively.

To distinguish living cells from dead cells, the samples were stained with propidium iodide immediately before the measurement. To achieve this, a final concentration of 0.1 µg/mL propidium iodide was added to the samples and incubated for 15 min at room temperature in the dark. The stained samples were analysed using a flow cytometer (BD Accuri™ C6, Becton, Dickinson and Company, Franklin Lakes, NJ, USA) equipped with four fluorescence detectors (FL1 533/30 nm, FL2 585/40 nm, FL3 > 670 nm, and FL4 675/25 nm), two scatter detectors, a blue laser (488 nm), and a red laser (640 nm). Deionised water was used as the sheath fluid. The instrument performance was monitored weekly with BDTM CS&T RUO Beads. The threshold settings FSC-H 500 and FL1-H 500, a limit of 25 μL 200.000 cells and the medium flow rate of 35 μL/min were used for the analysis of the samples, respectively.

The FSC-H signals were used to evaluate the total number of events in a sample. A pure media sample was also measured, and background counts were determined. The value of the forward scatter was used to select desired cell counts. Colour compensation was used to distinguish between the populations. The FL1-A signal was used to evaluate the GFP-producing cells within the sample. The FL2 signal was used to evaluate the dead cells within the sample (Figure S1).

## 3. Results

Our objective was to develop an easy-to-implement liquid clone selection method that makes use of an already-installed automated microbioreactor system. The approach aimed to streamline the strain construction process in automated platforms by reducing the reliance on multiple, often costly instruments. Hence, the approach was deemed particularly well-suited for academic settings, only requiring the basic infrastructure of a biofoundry. Similar to sophisticated colony-picking robots, the ALCS approach aims to achieve high selectivity and enables the immediate use of the gated strains in follow-up applications. Supported by a model-based set-up we showed the practical suitability with freshly transformed *E. coli* pJOE4056.2. Pre-assembled plasmids were used as we consider DNA assembly as a robust technique [20,21] (Supporting Information S2). Thereon, further studies encompassing different bacterial chassis were performed.

### 3.1. Preliminary Growth Investigation of Transformed Cells on Agar Plates

Following a common practice, clones are selected using agar plates with antibiotics as selection markers. Accordingly, we applied this procedure as a reference. Growth rates and generation times of transformed cells were monitored on solid plates. The generation times of the three different microorganisms are presented in Table 2 and used for the following model-based set-up of ALCS.

### 3.2. Integrating a Growth Model to Establish the ALCS Set-Up

**Figure 1:**
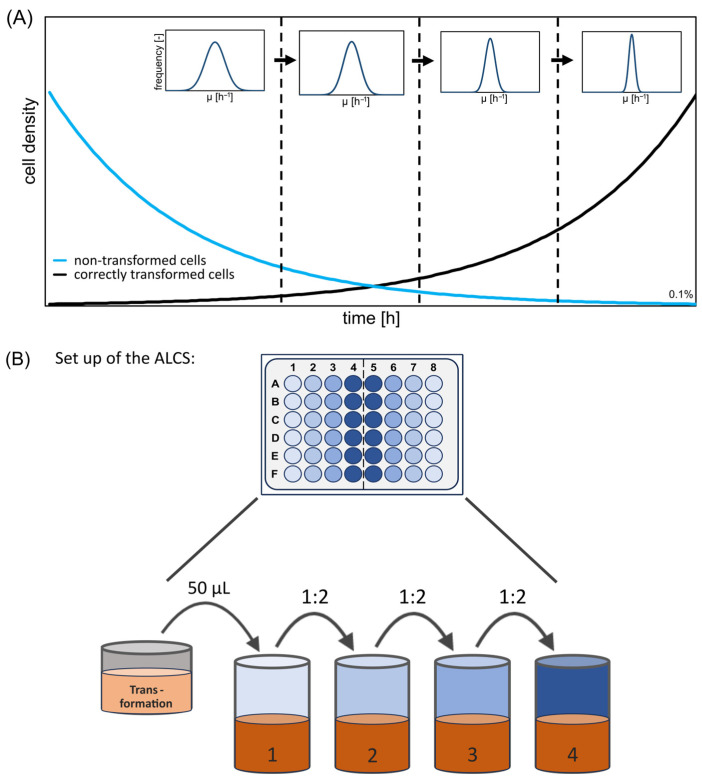
Schematic representation of the automated liquid-clone selection (ALCS). (**A**) ALCS achieves uniform specific growth rates of transformed cells by selectively promoting their growth (blue) while suppressing non-transformed cells (black). Over time, the dispersion of the Gaussian distribution of growth rates in the mixed culture decreases, resulting in uniform growth rates of transformed cells. (**B**) The flower plate is divided into two identical sections. ALCS is conducted in each row of these sections. Initially, 50 µL of the transformation approach is inoculated into the first well. After one (or two in the first step) generation time, the next well is inoculated from the first well using a 1:2 volumetric dilution.

To fulfil our objective of developing an easy-to-implement liquid clone selection method, we designed a model-based set-up. The uniform growth behaviour of the correctly transformed cells within the ALCS was the primary section criterion for clone selection. Notably, successful transformation provides a growth benefit as the plasmids contain antibiotic resistance genes (*amp^R^* or *kan^R^*) that enable growth in the presence of said antibiotics in the medium. Hence, transformed cells grow during ALCS, whereas non-transformed cells are suppressed (Figure 1). Coinciding with the increasing number of transformed cells, the growth rate of the population converges towards the growth rate of the correctly transformed cells. The Gaussian distributions of the growth rate become narrower and taller (Figure 1). To estimate the proper duration of the ALCS approach, the following assumptions were made (first, *E. coli* was considered):

- Input: The growth rate of transformed cells (1) was set to 0.25 h^−1^ (µ_1_), as calculated in the preliminary experiment. The non-survival rate of the non-transformed cells (2) was set to −0.7 h^−1^ (µ_2_).
- Input: The fraction cells f = c_x10_/c_x0_ is set—either via preliminary experiments or via estimations. *f* indicates the relative amount of transformed cells (c_x10_) based on the total amount of cells used for transformation (c_x0_). In this study, f was set to 0.0001 based on preliminary experiments. This was necessary to calculate the respective cultivation time.
- Target: The selection criteria (s) for the proportion of non-transformed cells in the end of the selection procedure was set to 0.02.

Based on the growth-related input parameters, we estimated the required cultivation time:

Given a batch culture (shaking flask, micro-well, microplate) the following equation (Equation (4)) holds true for the concentration c_x_ of non-transformed cells (indexed ‘2’) changing with the constant non-survival rate μ_2_:

(4) $$\frac{dc_{x2}}{dt}=\mu_{2}\cdot c_{x2}$$

Note, given that μ_2_ is negative because of the missing antibiotic resistance, the term describes the steady death of likewise cells in the culture. Accordingly, the cultivation time (Equation (5)) is derived as follows:

(5) $$t=\frac{1}{\mu_{2}}ln\left(\frac{c_{x2}}{c_{x20}}\right)$$

with c_x2_ as the final concentration of the non-transformed cells and c_x20_ as the related start concentration.

Considering the chosen selection criteria (s) of c_x2_ = 0.02 c_x1_ (with ‘1’ indexing the transformed cells) and further considering that the initial population c_x0_ comprises the sum of non-transformed (c_x20_) and transformed (c_x10_) cells (Equation (6)):

(6) $$c_{x10}{+c}_{x20}=c_{x0}$$

The necessary cultivation time t for obtaining the ratio of 50:1 transformed versus non-transformed cells (Equation (7)) equals to the following:

(7) $$t=\frac{1}{\mu_{2}}ln\left(\frac{0.02\cdot c_{x1}}{c_{x0}-c_{x10}}\right)$$

Preliminary measurements (or estimations) provide the ratio of transformed cells f = c_x10_/c_x0_. Accordingly, t may be calculated as follows:

(8) $$t=\frac{1}{\mu_{2}}ln\left(\frac{0.02}{1-f}\cdot f\cdot \frac{c_{x1}}{c_{x10}}\right)$$

Considering that:

(9) $$\frac{c_{x1}}{c_{x10}}=exp\left(\mu_{1}\cdot t\right)$$

*t* becomes the following:

(10) $$t=\frac{1}{\mu_{2}}ln\left(\frac{0.02}{1-f}\cdot f\cdot exp\left(\mu_{1}\cdot t\right)\right)=\frac{1}{\mu_{2}}ln\left(\frac{0.02\cdot f}{1-f}\right)+\frac{\mu_{1}}{\mu_{2}}\cdot t\;t=\frac{1}{\mu_{2}-\mu_{1}}ln\left(\frac{0.02\cdot f}{1-f}\right)$$

Next, the cultivation time was divided by the generation time of each organism to translate the model-based prediction into real cultivation times per well. As indicated in the Materials and Method section, generally one generation time was installed during ALCS except for the first well where one (or two) additional generation times were considered as lag-time equivalent. For slow-growing organisms, the lag phase should be determined via plate growth and incorporated into the design of ALCS for other organisms accordingly. The concept was transferred to the other organisms while µ_1_ and µ_2_ was adapted. In total, five generation times turned out to be sufficient for all tested organisms.

To facilitate advanced clone selection, a selection marker—in this case, antibiotics—was implemented to enhance the selection pressure. Additionally, liquid transfer was incorporated to introduce cell dilution. Both criteria allowed for the provision of a fresh medium during cultivation steps, thereby enhancing selection pressure and preventing nutrient limitation.

In this study, only induced cell growth and selection were executed to enable the precise evaluation of selection efficiency based on the fluorescent protein eGFP. To prevent false-positive selections (non-transformed cells), we incorporated liquid transfer and ensured the continuous presence of antibiotics in each well. To avoid false-negative clone selection, we monitored the selection process using eGFP expression and growth detection. In case of encountering issues with the ALCS approach, we have provided a trouble-shooting guide (Table S3).

The subsequent set-up of the ALCS method was designed. For inoculation, 50 µL of the transformation approach was added to the first well. The initial well underwent incubation for a duration equivalent to two generation times of the specific organism to facilitate regeneration after transformation. Based on the calculated cultivation time, three transfers were carried out to introduce fresh medium after every generation time interval. Each transfer involved a volume of 500 µL from the previous well and 500 µL fresh medium (dilution of 1:2). For an illustrative representation of the ALCS set-up, please refer to Figure 1. This workflow streamlines parallel clone selection, holding capacity up to twelve transformation approaches simultaneously (using a 48-well plate). The set-up was designed for easy scale-up to workflows that streamline 96 parallel clone selection using low-cost liquid-handling robots. The requirements for ALCS are limited to a liquid-handling robot equipped with a heat-shaker. Therefore, the implementation is simple for most biofoundries.

To ensure the success of the ALCS procedure, several factors are important to consider. Maintaining a sterile environment in the robotic system is crucial; therefore, the utilisation of sterile coverings like sealing foils and sterile cultivation conditions is essential. Proper calibration of the robotic pipetting system is essential for accurate trigger-related pipetting. Optimisation of cultivation settings, including temperature and shaking frequency, should be performed for the specific strain beforehand. Establishing and validating the transformation procedure for the organism in advance is necessary (Figure S3). Addressing these factors enhances the reliability and reproducibility of the ALCS procedure.

In the initial phase of validating the model-based concept, we implemented the ALCS with *E. coli* as the selected host organism. Subsequently, the ensuing section presents experimental data elucidating the successful transition of the ALCS to practical application.

### 3.3. Proof of Concept Clone Selection of E. coli pJOE4056.2

As described in the previous section, we established the ALCS for *E. coli* by applying the generation times specified in Table 2. Following a common practice, clones are selected using agar plates with antibiotics as selection markers. Accordingly, we applied this procedure as a reference. Growth rates and generation times of transformed cells were monitored on solid plates. The generation times of the three different microorganisms are presented in Table 2 and used for the following model-based set-up of ALCS.

**Figure 2:**
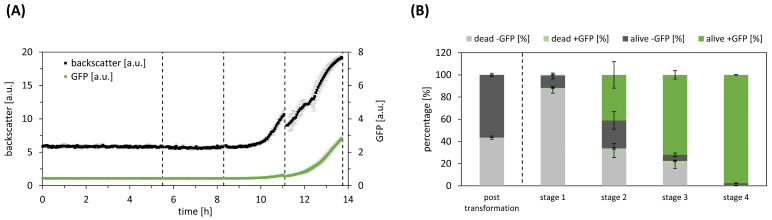
Automated liquid clone selection (ALCS) for *E. coli* pJOE4056.2. (**A**) Microbioreactor cultivation of ALCS, with growth curves (black) and GFP expression (green). Data represent mean of replicates (n = 6), with standard deviation as error bars. Dotted lines indicate liquid transfer. (**B**) Single-cell analysis results demonstrate deviation of cell types in the selection process of *E. coli* pJOE4056.2. Viable cells are categorised in transformed (green) and non-transformed (dark grey), based on GFP content. Non-viable cells are also categorised in transformed (light green) and non-transformed (light grey). Data represent mean of replicates (n = 6), with standard deviation as error bars.

Following manual heat shock transformation, the transformation approach was directly transferred to the automated platform and cultivated at 37 °C, using ampicillin as a selection marker. During the initial two cultivation stages, neither growth nor GFP production were detected. However, in the subsequent stages (stages three to four), both backscatter and GFP values exhibited an increase (Figure 2A). Following each stage, we analysed the cell population composition (S1), as depicted in Figure 2B. Immediately after transformation, less than 0.01 ± 0.01% of the cell population expressed the heterologous GFP, while almost 43 ± 1% of the cells had already died during the heat shock procedure. In the first stage, cells are mainly selected based on antibiotic resistance of transformed cells, resulting in 89 ± 3.4% of the cells being nonviable, and only 1 ± 0.5% of the viable cells expressing GFP. In subsequent steps, the percentage of GFP-expressing cells increased to 98 ± 0.2%. Notably, at this stage, the nonviable cells were effectively removed, leaving only 2 ± 0.2% of the cells still viable but devoid of GFP expression.

This initial proof of concept for the ALCS demonstrated successful implementation of the model-based configuration within the automated microbioreactor setting. The next step involves validating the model using experimental data.

### 3.4. Validation of Model Performance Using Experimental Data

**Figure 3:**
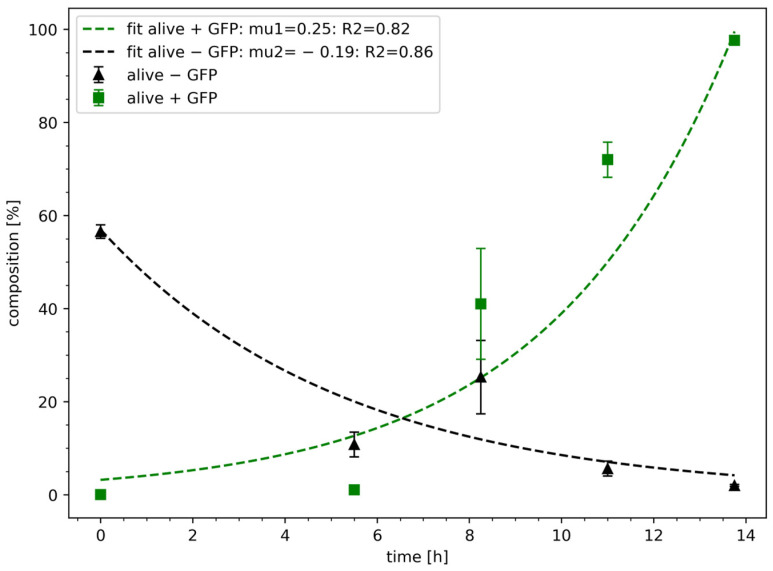
Comparison of growth model (dashed line) and experimental data (data points) of the automated liquid clone selection (ALCS). According to the model, composition of transformed cells (green) increases during the selection process, while non-transformed cells (black) are reduced. The values are presented as proportions of the different populations (%), considering only viable cells. Curve fitting, in which μ was set as an independent parameter, was carried out using Python and the Levenberg–Marquardt algorithm.

After the proof-of-concept study, the model was validated based on experimental data, assuming the non-survival behaviour as described earlier in Equation (4). The non-survival rate µ_2_ was fitted using the Levenberg–Marquardt algorithm, while the parameter c_x20_ was chosen based on experimental data (Figure 3). The parameter c_x10_ was estimated via Levenberg–Marquardt algorithm, while µ_1_ was set to 0.25 h^−1^. For the transformed cells (alive + GFP), the parameter c_x10_ resulted in 3.42%, which is higher than the experimental input data of 0.01 ± 0.01%. The curve fit has a significant R^2^ of 0.82. For the non-transformed cells (alive—GFP), the parameter µ_2_ resulted in −0.19 h^−1^, which is slower than assumed in the model-based calculation. Nevertheless, the curve fit is significant (R^2^ of 0.86).

The model can be used to design the ALCS and demonstrates significant curve fitting when compared to experimental data. This advancement prompted the subsequent phase, which involved assessing the robustness of the ALCS approach.

### 3.5. Further Investigation of the Clone Selection Method

Subsequent studies aimed to increase robustness of the ALCS method. Two key impacts were scrutinised: firstly, the influence of varying cell numbers at ALCS start, and secondly, the examination of the growth rate throughout the ALCS process.

**Figure 4:**
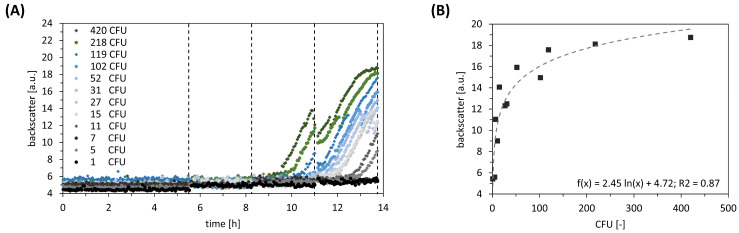
Dilution series of different initial colony-forming units (CFU) in the automated liquid clone selection (ALCS). Dotted lines indicate liquid transfer. (**A**) Growth curves of *E. coli* pJOE4056.2 (n = 1). (**B**) Correlation of backscatter value and CFU in the standard curve approach (R^2^ = 0.87).

For investigating the impact of transformed cells during the post-transformation stage, we executed a dilution series. Correlated backscatter values were compared with the CFU enumerated from agar plates (Figure 4A). It turned out that the minimum of seven transformed cells is needed to successfully apply ALCS. On the upper end, the maximum of 120 transformed cells should be considered for preventing non-wanted nutrient limitation that biases results. Additionally, we conducted a comparative analysis of the backscatter values at the end with the CFU counts. We identified a slight logarithmic correlation between backscatter and CFU (Figure 4B).

The core criterion of ALCS is the harmonisation of the population growth of the transformed cells. Accordingly, we examined the growth rate distributions at various stages (Table 3). As predicted, the average of population growth increased over time, while the standard deviation (SD) reduced. The SD was only considered when the growth rate was above 0.01, resulting in an increase in population homogeneity over the stages. In other words, population homogeneity increased during ALCS.

### 3.6. Successful Integration of Other Chassis to the Clone Selection Method

For challenging the universality of ALCS, different hosts were evaluated. The applicability of heat shock transformation, commonly employed in biofoundries, was a selection criterion for deciding on additional hosts. Accordingly, we chose *P. putida* as a Gram-negative showcase. Additionally, *C. glutamicum* was selected as a Gram-positive example, although heat shock plasmid transfer could not be applied for this host. Cultivation times for both organisms were adjusted, assuming that growth ratios between the non-transformed (indexed ‘2’) and transformed (indexed ‘1’) cells are equal to the observation with *E. coli* (Equation (11)). Hence, the correlation factor (k) −2.8 of *E. coli* was deemed to be valid for *P. putida* and *C. glutamicum*:

(11) $$k=\frac{{µ}_{2}}{{µ}_{1}}$$

**Figure 5:**
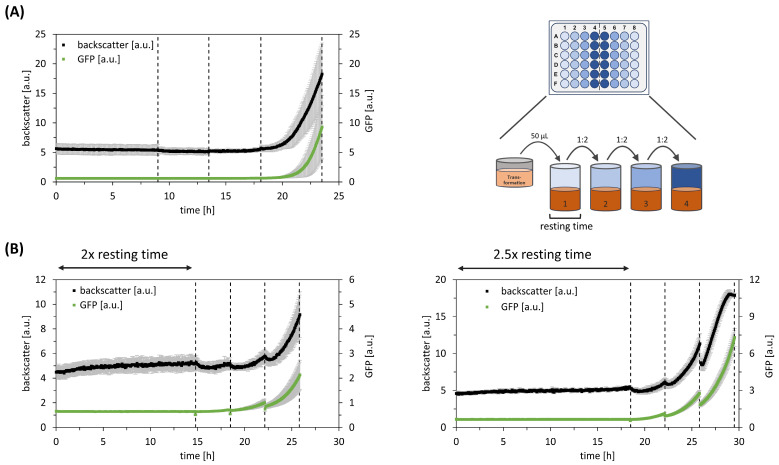
Automated liquid clone selection (ALCS) for *P. putida* pWN210 and *C. glutamicum* pJOE7706.2. Dotted lines indicate liquid transfer. (**A**) Microbioreactor cultivation of P. putida pWN210 ALCS, with growth curves (black) and GFP expression (green). Data represent mean of replicates (n = 12), with standard deviation as error bars. (**B**) Microbioreactor cultivation of *C. glutamicum* pJOE7706.2 ALCS, with growth curves (black) and GFP expression (green). Data represent mean of replicates (n = 6), with standard deviation as error bars. Resting time was included in the first well of ALCS. The left graph shows the addition of two resting times, while the right graph shows the addition of 2.5 resting times. One resting time represents one generation time.

*P. putida* underwent heat shock transformation, harbouring the plasmid pWN210 for GFP expression. The calculated generation time for *P. putida* was more than 1.5 times longer than *E. coli*. As described previously, clones were selected using solid agar plates with antibiotics as selection markers. The growth rates and generation times of transformed cells were monitored. The generation times of the three different micro-organisms are presented in Table 2 and used for the following model-based set-up of ALCS. Accordingly, the ALCS cultivation time was calculated as 23.75 h, but five generation times were still sufficient to enable clone selection. Although no growth could be observed within the first three stages (Figure 5), the final stage revealed the targeted increase in both backscatter and GFP expression. Concomitantly, the SD decreased. This demonstrates the successful application of ALCS with *P. putida*.

Using *C. glutamicum,* plasmid transformation was performed via electroporation. Furthermore, the BHI growth medium was chosen for clone selection. As the first ALCS application failed, an additional resting time in the first stage was considered in the follow-up study. The decision for the additional resting time was also motivated by independent observations of *C. glutamicum* on the agar plate, which showed the same trend. Figure 4 reveals that doubling the duration of the first stage yielded a growth pattern similar to what we had observed with *E. coli*. However, increasing the resting time by 2.5 times resulted in growth limitations towards the end of the selection process (Figure 5). Therefore, we decided on the doubled resting time when using *C. glutamicum* as a host.

At that point, we successfully incorporated both *P. putida* and *C. glutamicum* into our clone selection method. The integration of a resting time was a strategic adjustment addressing the special needs of *C. glutamicum*.

These results demonstrate our ability to monitor the growth of freshly transformed cells on agar plates and calculate the generation time accurately. Using this generation time as a basis, we established a workflow for liquid-based clone selection by applying the automated microbioreactor system. In our initial investigation with *E. coli* pJOE4056.2, we achieved a final purity of 98% correctly transformed cells at the conclusion of our clone selection process. Importantly, this method is also applicable to other microorganisms, including *C. glutamicum* and *P. putida*.

## 4. Discussion

ALCS represents a straightforward approach for clone selection. Basic modelling assumptions and the automation of protocols are showcased by using *E. coli*. Furthermore, the robustness of the approach was investigated for varying initial cell numbers and growth rates. Ultimately, other hosts could be successfully applied too.

The ALCS approach requires different ad initio input parameters, such as growth rates of transformed and non-transformed cells and relative amounts of transformed cells (*f*). For the latter, flow cytometry was applied, whereas growth rates were determined by measuring the increased size of CFUs. Those rates were lower than growth rates observed in liquid media [44], which is in agreement with the intrinsically limited 2-dimensional growth analysis using plates [45]. Assuming that the CFU on the agar plate have the same number of cell layers at time point 1 and time point 2, the above analysis method can be described as shown in Equation (1). If the culture is assumed to grow faster, µ will be estimated too low, leading to a conservative implementation of the approach. Therefore, increased division rates even improve selection. Nevertheless, CFU estimates could be well-applied to establish ALCS.

To achieve the population of 98% correctly transformed cells, fresh ampicillin was supplemented at each stage, selecting for the antibiotic-resistant cells. The fact that 2% of cells apparently did not produce GFP may also be explained by the missing detection quality of correctly transformed freshly divided cells that did not grow yet [46].

The non-linear estimation of *E. coli* growth rates was performed by the Levenberg–Marquardt algorithm, achieving acceptable R^2^ values of 0.82 and 0.86 for µ_1_ and µ_2_, respectively. However, the likewise-estimated non-survival rate (µ_2_) was slower than the initial estimate. For instance, [47] reported non-survival rates of about −0.5 h^−1^. Likely, the discrepancy mirrors the model-based overestimated proportion of initially transformed cells (c_x10_) of 3.42%.

During ALCS, cellular growth was quantified via backscatter measurements, while the expression of GFP was assessed via fluorescence. Although the backscatter detection limit was expected between OD_600_ 0.2 and 0.6 [48], the use of complex medium increased the lower limit of OD_600_ from 0.5 to 1. In these cases, the application of flow cytometry compensated our measurements.

While investigating the robustness of ALCS, we found two key impacts: firstly, the influence of varying cell numbers at ALCS start, and secondly, the increasing homogeneity during ALCS. The first was investigated by executing dilution series: At the lower end of 7 CFU, the transformed cells undergo further dilution, which is likely to limit growth because of the poor availability of dissolved CO_2_/HCO_3_^−^ for anaplerotic reactions. On the contrary, high initial CFUs cause stationary backscatter values at the end of the selection, mimicking limited oxygen availability. Consequently, harvesting during exponential growth becomes impossible, which biases the standardised procedure for glycerol stocks formation. A logarithmic correlation between final backscatter values and cell numbers at the ALCS start has been observed (R^2^ = 0.87).

The second impact factor, namely the culture homogeneity, is characterised by the standard deviation of the growth distribution. The example of *E. coli* (Table 3) showcases how SD converges from 155% (stage 1) to 11% (stage 4). Notably, the trend mirrors the number of correctly transformed cells (Figure 3), which confirms the ALCS approach.

To showcase the universality of ALCS, additional Gram-negative (*P. putida*) and Gram-positive (*C. glutamicum*) prokaryotes were tested. The latter requires electroporation for DNA transformation instead of the heat shock protocol used for the Gram-negative microbes. For simplification, the correlation factor *k* = −2.8 was introduced, linking growth rates between transformed and non-transformed cells. The factor was quantified based on the initial experience with *E. coli*. Although, the suitability was demonstrated in the current studies, further fine tuning may be necessary for other microbes.

Though ALCS could be successfully demonstrated for *P. putida,* particularities occurred: Significant growth was only observed in the final stage, which gives rise to the assumption that lag phases were much longer than for *E. coli*. Apparently, the cells required more time for adaption, which was not observable on agar plates. Nevertheless, clone selection succeeded, which underlines the robustness of the approach, despite the weakness to translate 2D growth observations into micro wells.

*C. glutamicum* required electroporation for plasmid transformation, which was performed manually. However, automated protocols exist [49]. Like *P. putida*, *C. glutamicum* demanded extended lag phases compared to agar plates. Pondering the need to extend lag phases versus too-high cell titres at the end of ALCS, the compromise of doubled resting times in the first well was chosen, which finally provided satisfactory results.

Despite the adjustments needed, the successful implementation of ALCS could be shown for *P. putida* and *C. glutamicum*. In both cases, the standard deviations of the growth rates converged, indicating that strain selection succeeded.

Some biofoundries apply colony-picking stations for clone selection, as demonstrated by various studies [19,25,26,27,28]. Integrating a separate colony-picking station into the platform expands the number of devices, resulting in increased capital expenditure. Some customised solutions, using a combination of liquid-handling robots and CCD cameras, have been proposed [32,33]. The complexity of the colony-picking method rises if solid agar plates and incubators are required for cultivation.

Jian et al. (2022) introduced the development of a single-cell microliter-droplet screening system, known as MISS Cell, designed for clone selection and cultivation [36]. This system involves the construction of a microfluidic device, enabling single-cell separation. While MISS Cell offers a cost-effective and highly selective solution, it remains a separate system that must be integrated into the platform. One limitation of the method is the lack of demonstrated use of MISS Cell for individual high-throughput transformation approaches.

An alternative approach for clone selection applies a flow cytometry device equipped with a cell-sorting system [35]. However, proper cell sorting requires the implementation of a biosensor, which may not always be available. Moreover, polyclonal screening of transformed cells may be applied [5]. Apparently, this method lacks selectivity; although, it favours selection speed.

On contrary to those approached, ALCS does not need additional devices. ALCS is based on liquid handling, which simplifies the procedure compared to agar selection, as different heights of agar, low contrast of colonies, etc., do not occur. ALCS did not require sophisticated biosensors, which, themselves, might bias the cellular performance. While others have implemented a polyclonal screening approach in their biofoundry, [5,6,34] our ALCS offers higher selectivity. The proposed ALCS is best suited for two-step cloning procedures that start with plasmid construction using a simple cloning host such as *E. coli* DH5α and continue with the construction of production strains [50]. Another application is the initial selection in a library preparation approach when correctly transformed cells need to be selected, and different strains must be separated. This could be performed as a follow up to ALCS [51].

The suggested ALCS workflow allows for the parallel execution of twelve clone selections per 48-well plate but could be easily scaled, e.g., to 24 clones in 96-well plates or 96 clones in 384-well plates. Related clone-testing capacities may be installed by low-cost and multi-functional liquid-handling robots, such as OT-2 robots (Opentrons, New York, NY, USA), which are already commonly accepted in academic labs. 

In the main studies, pre-assembled and purified plasmids were applied. As previously mentioned, modern molecular biology techniques are robust and show a minimum error rate [20,21]. We could show that this also holds true for the DNA assembly used in the cloning workflow in our semi-automated platform (Figure S2). Hence, an equally homogeneously transformed population with minimised genotypic variation was selected during ALCS.

Up to now, only plasmids expressing GFP were applied in this study. The selection of strains expressing functional genes should work in a similar way, as this method does not rely on fluorescence measurement. By analogy, additional hosts should be tested with our ALCS approach. Nevertheless, this work opens avenues for further research, including the method integration into specific biofoundry platforms and the incorporation of additional organisms to broaden its applicability.

## Figures and Tables

**Table 1:** Plasmids used in this study.

Plasmid	Plasmid Characteristics	Reference
pJOE 4056.2	P_T7_/P_rhaB_; Amp^R^; ori pBR322; eGFP; N-terminal 6xHis	[37]
pJOE 7706.1	P_tac_; Kan^R^; ori pBR322; eGFP; N-terminal 6xHis	[38]
pWN 210	P_Bba_J23101_; Kan^R^; ori pBBR1; sfGFP; N-terminal 6xHis	[39]

**Table 2:** Growth rate and generation time of *E. coli* pJOE4056.2 (n = 7), *P. putida* pWN210 (n = 7), and *C. glutamicum* pJOE7706.2 (n = 9) on agar plates. Agar plates were incubated at 37 °C or 30 °C for 25 to 69 h.

	*E. coli * pJOE4056.2	*P. putida * pWN210	*C. glutamicum* pJOE7706.2
Growth rate [h^−1^]	0.25 ± 0.01	0.15 ± 0.027	0.19 ± 0.015
Generation time [h]	2.75 ± 0.07	4.72 ± 0.8	0.69 ± 0.31

**Table 3:** Growth rates and standard deviation of *E. coli* pJOE4056.2, *P. putida* pWN210 and *C. glutamicum* pJOE7706.2 (n = 6) in automated liquid clone selection (ALCS).

		Stage 1	Stage 2	Stage 3	Stage 4
*E. coli* pJOE4056.2	Growth rate [h^−1^]	0.00	0.01	0.17	0.30
	Standard deviation [%]	155	164	39	11
*P. putida* pWN210	Growth rate [h^−1^]	0.00	0.01	0.01	0.21
	Standard deviation [%]	71	65	121	33
*C. glutamicum* pJOE7706.2	Growth rate [h^−1^]	0.01	0.01	0.04	0.13
	Standard deviation [%]	44	325	42	26

## Data Availability

Raw data and results are publicly accessible in our DaRUS repository (https://doi.org/10.18419/darus-4355) accessed on 2 August 2024. Please contact the corresponding author for further information.

## Supplementary Materials

The following supporting information can be downloaded at: https://www.mdpi.com/article/10.3390/bioengineering11090892/s1, Supporting Information S1: Flow cytometer analysis; Supporting Information S2: Evaluation of assembled plasmids [20,21,52]; Supporting Information S3: Optimising transformation efficiency; Supporting Information S4: Troubleshooting guide.

## References

[1] Carbonell P., Jervis A.J., Robinson C.J., Yan C., Dunstan M., Swainston N., Vinaixa M., Hollywood K.A., Currin A., Rattray N.J.W. (2018). An automated Design-Build-Test-Learn pipeline for enhanced microbial production of fine chemicals. Commun. Biol..

[2] Chao R., Mishra S., Si T., Zhao H. (2017). Engineering biological systems using automated biofoundries. Metab. Eng..

[3] Ko S.C., Cho M., Lee H.J., Woo H.M. (2022). Biofoundry Palette: Planning-Assistant Software for Liquid Handler-Based Experimentation and Operation in the Biofoundry Workflow. ACS Synth. Biol..

[4] Johnson J.R., D’Amore R., Thain S.C., Craig T., McCue H.V., Hertz-Fowler C., Hall N., Hall A.J.W. (2016). GeneMill: A 21st century platform for innovation. Biochem. Soc. Trans..

[5] Chao R., Liang J., Tasan I., Si T., Ju L., Zhao H. (2017). Fully Automated One-Step Synthesis of Single-Transcript TALEN Pairs Using a Biological Foundry. ACS Synth. Biol..

[6] Si T., Chao R., Min Y., Wu Y., Ren W., Zhao H. (2017). Automated multiplex genome-scale engineering in yeast. Nat. Commun..

[7] Bryant J.A., Kellinger M., Longmire C., Miller R., Wright R.C. (2023). AssemblyTron: Flexible automation of DNA assembly with Opentrons OT-2 lab robots. Synth. Biol..

[8] Steel H., Habgood R., Kelly C., Papachristodoulou A. (2019). Chi. Bio: An open-source automated experimental platform for biological science research. BioRxiv.

[9] Janzen N.H., Striedner G., Jarmer J., Voigtmann M., Abad S., Reinisch D. (2019). Implementation of a Fully Automated Microbial Cultivation Platform for Strain and Process Screening. Biotechnol. J..

[10] Krausch N., Kim J.W., Barz T., Lucia S., Groß S., Huber M.C., Schiller S.M., Neubauer P., Cruz Bournazou M.N. (2022). High-throughput screening of optimal process conditions using model predictive control. Biotechnol. Bioeng..

[11] Radivojević T., Costello Z., Workman K., Garcia Martin H. (2020). A machine learning Automated Recommendation Tool for synthetic biology. Nat. Commun..

[12] Haby B., Hans S., Anane E., Sawatzki A., Krausch N., Neubauer P., Cruz Bournazou M.N. (2019). Integrated Robotic Mini Bioreactor Platform for Automated, Parallel Microbial Cultivation with Online Data Handling and Process Control. SLAS Technol..

[13] Holowko M.B., Frow E.K., Reid J.C., Rourke M., Vickers C.E. (2021). Building a biofoundry. Synth. Biol..

[14] Carbonell P., Currin A., Dunstan M., Fellows D., Jervis A., Rattray N.J.W., Robinson C.J., Swainston N., Vinaixa M., Williams A. (2016). SYNBIOCHEM-a SynBio foundry for the biosynthesis and sustainable production of fine and speciality chemicals. Biochem. Soc. Trans..

[15] Hillson N., Caddick M., Cai Y., Carrasco J.A., Chang M.W., Curach N.C., Bell D.J., Le Feuvre R., Friedman D.C., Fu X. (2019). Building a global alliance of biofoundries. Nat. Commun..

[16] Kelly J. (2012). The organism is the product. ACS Synth. Biol..

[17] Hill P., Benjamin K., Bhattacharjee B., Garcia F., Leng J., Liu C.-L., Murarka A., Pitera D., Rodriguez Porcel E.M., Da Silva I. (2020). Clean manufacturing powered by biology: How Amyris has deployed technology and aims to do it better. J. Ind. Microbiol. Biotechnol..

[18] Wehrs M., de Beaumont-Felt A., Goranov A., Harrigan P., de Kok S., Lieder S., Vallandingham J., Tyner K. (2020). You get what you screen for: On the value of fermentation characterization in high-throughput strain improvements in industrial settings. J. Ind. Microbiol. Biotechnol..

[19] Kang D.H., Ko S.C., Heo Y.B., Lee H.J., Woo H.M. (2022). RoboMoClo: A Robotics-Assisted Modular Cloning Framework for Multiple Gene Assembly in Biofoundry. ACS Synth. Biol..

[20] Dodd T., Botto M., Paul F., Fernandez-Leiro R., Lamers M.H., Ivanov I. (2020). Polymerization and editing modes of a high-fidelity DNA polymerase are linked by a well-defined path. Nat. Commun..

[21] Engler C., Kandzia R., Marillonnet S. (2008). A one pot, one step, precision cloning method with high throughput capability. PLoS ONE.

[22] Robinson C.J., Carbonell P., Jervis A.J., Yan C., Hollywood K.A., Dunstan M.S., Currin A., Swainston N., Spiess R., Taylor S. (2020). Rapid prototyping of microbial production strains for the biomanufacture of potential materials monomers. Metab. Eng..

[23] Xia M., Yan X., Zan Z., Yang F., Liu M., Xue D., Shen Y., Wang M. (2022). Construction of automated high-throughput screening method for finding efficient 3-ketosteroid 1,2-dehydrogenating strains. Appl. Microbiol. Biotechnol..

[24] Tenhaef N., Stella R., Frunzke J., Noack S. (2021). Automated Rational Strain Construction Based on High-Throughput Conjugation. ACS Synth. Biol..

[25] Bairy S., Gopalan L.N., Setty T.G., Srinivasachari S., Manjunath L., Kumar J.P., Guntupalli S.R., Bose S., Nayak V., Ghosh S. (2018). Automation aided optimization of cloning, expression and purification of enzymes of the bacterial sialic acid catabolic and sialylation pathways enzymes for structural studies. Microb. Biotechnol..

[26] Moffat A.D., Elliston A., Patron N.J., Truman A.W., Carrasco Lopez J.A. (2021). A biofoundry workflow for the identification of genetic determinants of microbial growth inhibition. Synth. Biol..

[27] Dörr M., Fibinger M.P.C., Last D., Schmidt S., Santos-Aberturas J., Böttcher D., Hummel A., Vickers C., Voss M., Bornscheuer U.T. (2016). Fully automatized high-throughput enzyme library screening using a robotic platform. Biotechnol. Bioeng..

[28] Chow J.Y., Shi Choo K.L., Lim Y.P., Ling L.H., Truc Nguyen G.K., Xue B., Chua N.H., Yew W.S. (2021). Scalable Workflow for Green Manufacturing: Discovery of Bacterial Lipases for Biodiesel Production. ACS Sustain. Chem. Eng..

[29] Hansen A.D., Pollard M.J., Searles W.L., Uber D.C., Jaklevic J.M. (1993). A High-Speed Automated Colony Picking Machine.

[30] Jones P., Watson A., Davies M., Stubbings S. (1992). Integration of image analysis and robotics into a fully automated colony picking and plate handling system. Nucleic Acids Res..

[31] Huang C., He K., Liu C., Fu X., Du R. A Colony Picking Robot with Multi-Pin Synchronous Manipulator. Proceedings of the 2018 IEEE International Conference on Information and Automation (ICIA).

[32] Del Olmo Lianes I., Yubero P., Gómez-Luengo Á., Nogales J., Espeso D.R. (2023). Technical upgrade of an open-source liquid handler to support bacterial colony screening. Front. Bioeng. Biotechnol..

[33] Hartley T., Stewart C., Stewart R., Munroe D.J. (2009). Cost-Effective Addition of High-Throughput Colony Picking Capability to a Standard Liquid-Handling Platform. JALA J. Assoc. Lab. Autom..

[34] Liang J., Chao R., Abil Z., Bao Z., Zhao H. (2014). FairyTALE: A high-throughput TAL effector synthesis platform. ACS Synth. Biol..

[35] Jha R.K., Kern T.L., Fox D.T., M Strauss C.E. (2014). Engineering an Acinetobacter regulon for biosensing and high-throughput enzyme screening in *E. coli* via flow cytometry. Nucleic Acids Res..

[36] Jian X., Guo X., Cai Z., Wei L., Wang L., Xing X.-H., Zhang C. (2022). Single-cell microliter-droplet screening system (MISS Cell): An integrated platform for automated high-throughput microbial monoclonal cultivation and picking. Biotechnol. Bioeng..

[37] Wegerer A., Sun T., Altenbuchner J. (2008). Optimization of an *E. coli* L-rhamnose-inducible expression vector: Test of various genetic module combinations. BMC Biotechnol..

[38] Hoffmann J., Altenbuchner J. (2014). Hyaluronic acid production with Corynebacterium glutamicum: Effect of media composition on yield and molecular weight. J. Appl. Microbiol..

[39] Venkataraman M., Yñigez-Gutierrez A., Infante V., MacIntyre A., Fernandes-Júnior P.I., Ané J.-M., Pfleger B. (2023). Synthetic Biology Toolbox for Nitrogen-Fixing Soil Microbes. ACS Synth. Biol..

[40] Schindelin J., Arganda-Carreras I., Frise E., Kaynig V., Longair M., Pietzsch T., Preibisch S., Rueden C., Saalfeld S., Schmid B. (2012). Fiji: An open-source platform for biological-image analysis. Nat. Methods.

[41] Neidhardt F.C., Curtiss R. (1996). Escherichia coli and Salmonella: Cellular and Molecular Biology.

[42] Davis M.L., Mounteer L.C., Stevens L.K., Miller C.D., Zhou A. (2011). 2D motility tracking of Pseudomonas putida KT2440 in growth phases using video microscopy. J. Biosci. Bioeng..

[43] Schubert K., Sieger B., Meyer F., Giacomelli G., Böhm K., Rieblinger A., Lindenthal L., Sachs N., Wanner G., Bramkamp M. (2017). The Antituberculosis Drug Ethambutol Selectively Blocks Apical Growth in CMN Group Bacteria. mBio.

[44] Bipatnath M., Dennis P.P., Bremer H. (1998). Initiation and velocity of chromosome replication in *Escherichia coli* B/r and K-12. J. Bacteriol..

[45] Alalam H., Graf F.E., Palm M., Abadikhah M., Zackrisson M., Boström J., Fransson A., Hadjineophytou C., Persson L., Stenberg S. (2020). A High-Throughput Method for Screening for Genes Controlling Bacterial Conjugation of Antibiotic Resistance. mSystems.

[46] Siegele D.A., Hu J.C. (1997). Gene expression from plasmids containing the araBAD promoter at subsaturating inducer concentrations represents mixed populations. Proc. Natl. Acad. Sci. USA.

[47] Yourassowsky E., van der Linden M.P., Crokaert F. (1989). Correlation between growth curves and killing curves of *Escherichia coli* in the presence of fleroxacin and ampicillin. Chemotherapy.

[48] Kensy F., Zang E., Faulhammer C., Tan R.-K., Büchs J. (2009). Validation of a high-throughput fermentation system based on online monitoring of biomass and fluorescence in continuously shaken microtiter plates. Microb. Cell Fact..

[49] Bae C., Butler P.J. (2006). Automated single-cell electroporation. BioTechniques.

[50] Guo E., Fu L., Fang X., Xie W., Li K., Zhang Z., Hong Z., Si T. (2022). Robotic Construction and Screening of Lanthipeptide Variant Libraries in *Escherichia coli*. ACS Synth. Biol..

[51] HamediRad M., Chao R., Weisberg S., Lian J., Sinha S., Zhao H. (2019). Towards a fully automated algorithm driven platform for biosystems design. Nat. Commun..

[52] Guitart Font E., Sprenger G.A. (2020). Opening a Novel Biosynthetic Pathway to Dihydroxyacetone and Glycerol in Escherichia coli Mutants through Expression of a Gene Variant (fsaAA129S) for Fructose 6-Phosphate Aldolase. Int. J. Mol. Sci..

