# Supplementary material for: Getting the Right Clones in an Automated Manner: An Alternative to Sophisticated Colony-Picking Robotics
Source: Bioengineering (Basel). 2024 Sep 3;11(9):892. doi: 10.3390/bioengineering11090892 (PMC11429294; doi:10.3390/bioengineering11090892)
Supplement: Supplementary file 1 [file bioengineering-11-00892-s001.zip › bioengineering-3156036-supplementary.pdf]

Supporting Information S1: Flow cytometer analysis

A flow cytometry analysis of ALCS was used to distinguish between four different populations. While living and dead cells were distinguished via propidium iodide staining, GFP expression was detected via green fluorescence signal. A clear separation between the populations was possible not only in the control sample (Figure S1A) but also in the ALCS sample at the final stage (Figure S1B).

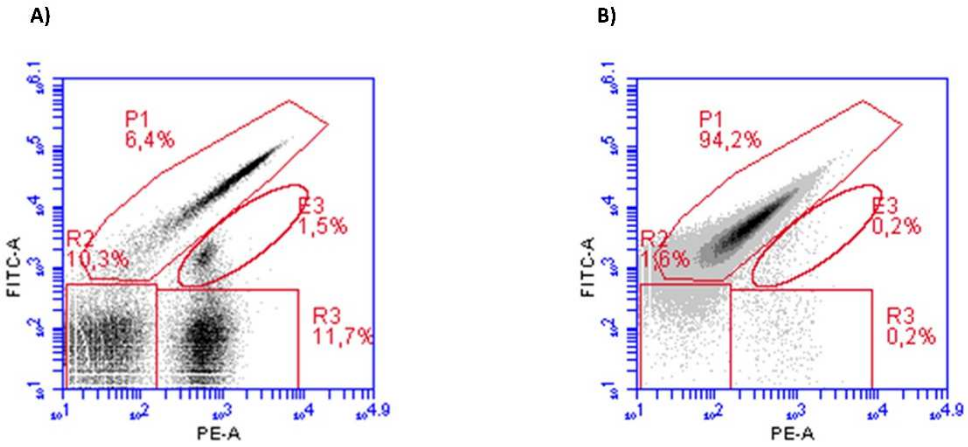

Figure S1: Flow cytometry analysis of *Escherichia coli* BL21(DE3) pJOE4056.2, evaluated for viability (propidium iodide staining) and GFP expression. In total, 200,000 events/sample (gated on PE-A vs FITC-A scatter) were acquired on a flow cytometer (BD Accuri^{TM} C6) with FSC-H 500 and FL1-H 500. Results show propidium iodide staining (PE-A) vs. GFP (FITC-A) log–scale dot plots for A): A control sample prepared by mixing live and ethanol-killed transformed and non-transformed cells, and B): An exemplary sample from the final stage of the ALCS process. Gates were drawn according to the four populations visible in the control sample: PI-/GFP- (alive/non-expressing, R2); PI+GFP- (dead/non-expressing, R3); PI-/GFP+ (alive/expressing, P1); and PI+/GFP+ (dead/ expressing, E3).

Supporting Information S2: Evaluation of assembled plasmids

Modern molecular biology techniques are robust and have a minimal error rate, which has been demonstrated in several papers [20,21]. To investigate whether this is also true for our in-house cloning workflow using Gibson assembly, the following experiments were performed. The plasmid pJNTN_GFP was assembled from the existing pJOE4056.2 and analysed.

Materials and Methods

DNA assembly

DNA parts for Gibson assembly were generated via PCR reaction using hot start Q5 DNA polymerase according to the manufacturing protocol (NEB, Ipswich, USA) and related primers (Table S1). The backbone pJNTN-m-L was linearized using the NdeI and dephosphorylated using arctic phosphatase according to the manufacturing protocol (NEB, Ipswich, USA). The plasmid pJNTN_GFP was assembled by Gibson assembly using NEBuilder HiFi DNA assembly Master Mix according to the manufacturer protocol (NEB, Ipswich, USA).

Table S1: Primer used in this study.

**Oligonucleotide**	**Sequence (5'-> 3')**	**function**
**GFP_pJN_fwd**	<u>AATTTCACACAGGAGATATACA</u>ATGACCATGATTACGCATCATCATCATCATCATG	forward primer, underline: the Gibson annealing side
**GFP_pJN_rev**	CCGGGTACCGAGCTCGACATTA<u>CTTGTACAGCTCGTCCATGCCG</u>	reverse primer, underline: the Gibson annealing side

The plasmids used in this study are listed in Table S2. Transformation and plating were conducted as already described in the main manuscript, but Kanamycin (50 µg/mL) was used as a selection marker.

Table S2: Plasmids used in this study.

**Plasmid**	**Plasmid characteristics**	**source**
**pJNTN-m-L**	P_{tac}; Kan^R; $lacI^q$	[52]
**pJNTN_GFP**	P_{tac}; Kan^R; $lacI^q$	This study

Cultivation in microplates

A flat-bottom microplate with 96 wells was filled each with 200 µL of 2xTY supplemented with 1 µM of IPTG and 50 ng/mL of kanamycin. Each well was inoculated with a single colony-forming unit from the agar plate which was cultivated overnight. The plate was cultivated for 24 h at 37 °C in a microplate reader (Tecan Spark, Tecan Trading AG, Männedorf, Schweiz), OD (600 nm), and fluorescence (Emission: 507 nm; Excitation: 488 nm) was measured every 10 min. The final measurement was used for evaluating cloning efficiency.

Results and Discussion

All 91 colony-forming units exhibited uniform growth and GFP expression, with maximum GFP values reaching 92.1 ± 7.4 [a.u.]. The experiment achieved a 100 % assembly efficiency (Figure S2). Strains containing pre-assembled plasmids can be seamlessly integrated into ALCS.

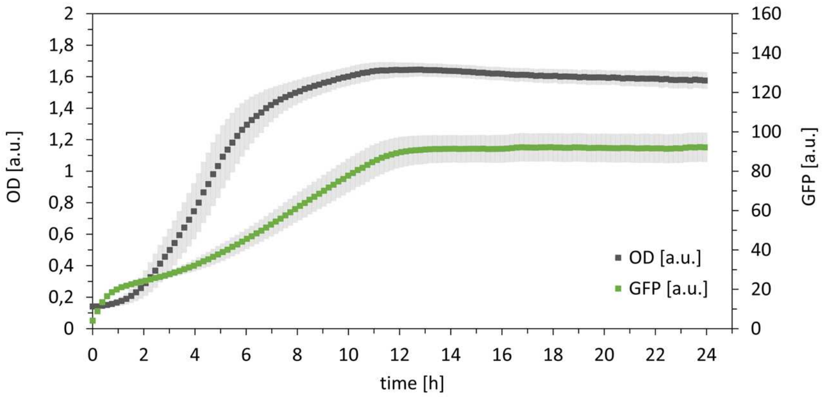

Figure S2: Cultivation of single colony-forming units of *E. coli* pJNTN_GFP. Microplate cultivation with growth curves (black) and GFP expression (green). Data represent mean of replicates (n=96), with standard deviation as error bars.

Supporting Information S3: Optimising transformation efficiency

A crucial element of the ALCS process is transformation efficiency. In order to assess the robustness of the method in this regard, we evaluated it based on transformation efficiency (Figure 4). Prior to establishing the ALCS process, we conducted comprehensive studies to enhance transformation efficiency. This allowed us to ascertain the optimal conditions for the ALCS process. This optimisation can serve as a reference point for implementing additional organisms.

Results and Discussion

When optimising transformation efficiency, several parameters have a significant impact (Figure S3). The use of a PCR MTP has been shown to result in an increase in transformation efficiency, as measured in colony-forming units (CFU). This may be due to improved heat transfer. Additionally, setting the heat shock parameter to 60 s and 42 °C could enhance the transformation efficiency. However, higher temperatures and extended exposure may induce elevated stress levels. Nevertheless, these parameters have only a minimal impact.

Switching to an automated workflow has the potential to significantly enhance the transformation efficiency. Furthermore, reducing the volume of the transformation approach has also demonstrated increased transformation efficiency. This may occur due to enhanced heat transfer.

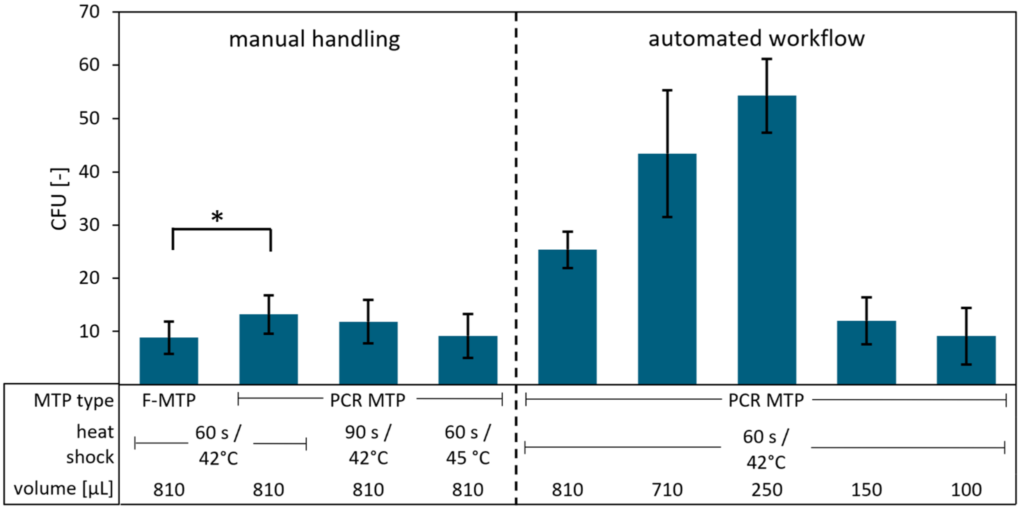

Figure S3: Optimisation of transformation efficiency. Manual and automated workflow were tested. Also, different MTP types were compared. The heat shock procedure was evaluated at different conditions of times and temperatures. The volume of transformation was also adjusted. The data represent the mean of eight replicates, with standard deviation as error bars. Abbreviations: CFU: colony-forming unit, MTP: microtiter plate, F-MTP: flat-bottom MTP, PCR MTP: polymerase chain reaction MTP. A two-tailed Student's t-test was used for statistical analysis (* = $p < 0.05$).

Supporting Information S4: Troubleshooting guide

When encountering issues with ALCS procedure, it is essential to have a structured approach to identify and resolve the problem efficiently. This troubleshooting guide has been developed to assist in the systematic diagnosis and resolution of common issues, ensuring minimal disruption to the workflow. By following the steps outlined below (Table S3), the root cause can be rapidly identified and an appropriate solution implemented.

Table S3: Troubleshooting guide for ALCS method.

No.	Common issues	Resolution
**1**	At the end of the ALCS procedure, there is no growth.	A) The cause of the problem could be low transformation efficiency. To solve this problem, the transformation efficiency can be improved, or a larger initial inoculation volume can be used.<br>B) The cause of the problem could be a long lag time. To solve this problem, an additional lag time could be implemented in the first stage to ensure adaptation to the media.
**2**	High false-negative clone selection.	The cause of the problem may be low selection pressure. Ensure effective selection pressure by adding sufficient antibiotics or fresh antibiotics at each step.
**3**	High false-positive clone selection.	The cause of the problem may be too much selection pressure. Add only a minimal but sufficient amount of antibiotics to the ALCS.
**4**	Unexpected turbidity of the culture and contamination.	A) The cause of the problem may be low selection pressure. Ensure effective selection pressure by adding sufficient antibiotics or fresh antibiotics at each step.<br>B) The cause of the problem may be technical cross-contamination. Ensure adequate washing and disinfection of fixed tips and single use of disposable tips.
**5**	There is a discrepancy between the calculated and actual volumes in the various wells.	A) The problem may be due to liquid transfer. The technical settings of the pipettes used may need to be checked and adjusted.<br>B) The problem may be due to evaporation. At high temperatures and low working volumes, the medium can evaporate and affect the ALCS process. A low evaporation sterile foil may help.
